# The Role of c-Abl Tyrosine Kinase in Brain and Its Pathologies

**DOI:** 10.3390/cells12162041

**Published:** 2023-08-10

**Authors:** Helena Motaln, Boris Rogelj

**Affiliations:** 1 Department of Biotechnology, Jozef Stefan Institute, 1000 Ljubljana, Slovenia; 2 Faculty of Chemistry and Chemical Technology, University of Ljubljana, 1000 Ljubljana, Slovenia; boris.rogelj@ijs.si

**Keywords:** c-Abl (Abelson) tyrosine kinase, neurodegeneration, Alzheimer’s disease, Parkinson’s disease, kinase signaling

## Abstract

Differentiated status, low regenerative capacity and complex signaling make neuronal tissues highly susceptible to translating an imbalance in cell homeostasis into cell death. The high rate of neurodegenerative diseases in the elderly population confirms this. The multiple and divergent signaling cascades downstream of the various stress triggers challenge researchers to identify the central components of the stress-induced signaling pathways that cause neurodegeneration. Because of their critical role in cell homeostasis, kinases have emerged as one of the key regulators. Among kinases, non-receptor tyrosine kinase (Abelson kinase) c-Abl appears to be involved in both the normal development of neural tissue and the development of neurodegenerative pathologies when abnormally expressed or activated. However, exactly how c-Abl mediates the progression of neurodegeneration remains largely unexplored. Here, we summarize recent findings on the involvement of c-Abl in normal and abnormal processes in nervous tissue, focusing on neurons, astrocytes and microglial cells, with particular reference to molecular events at the interface between stress signaling, DNA damage, and metabolic regulation. Because inhibition of c-Abl has neuroprotective effects and can prevent neuronal death, we believe that an integrated view of c-Abl signaling in neurodegeneration could lead to significantly improved treatment of the disease.

## 1. Introduction

Despite all the progress in understanding neurodegeneration, we are still unable to close the knowledge gap about what exactly happens in brain cells just before the onset of neurodegenerative diseases like Alzheimer’s disease (AD), Parkinson’s disease (PD), amyotrophic lateral sclerosis (ALS) or frontotemporal dementia (FTD). This is probably one of the reasons why there are still no effective treatments for all these diseases and why many targets identified so far have shown very limited neuroprotective effects in human studies [1]. Because current treatments are mostly symptomatic, researchers are urgently seeking novel neuroprotective agents and disease-modifying strategies that would slow or hopefully halt the progression of neurodegeneration altogether.

What several neurodegenerative diseases (ND) have in common is the death of various types of neurons, usually due to the formation of extracellular and/or intracellular protein inclusions that impair cellular processes, disbalance homeostasis and induce programmed cell death or apoptosis [1,2]. In many ND including ALS, the pathogenesis and death of motor neurons are also thought to be triggered by non-cell-autonomous mechanisms, since the conditioned medium from SOD1-mutant primary mouse astrocytes has been shown to cause death of exposed cultured motor neurons in vitro [3]. Moreover, the strong correlation between cognitive decline and synapse loss in several ND supports the idea that synaptic damage may indeed be one of the main pathogenic mechanisms underlying the development and progression of neurodegeneration [4]. However, considering that in AD, PD and FTD, in addition to the observed synaptic loss, defects in neurotransmitter activity, signaling efficiency, damage/repair systems, cell cycle, glial function, and neuroinflammatory processes have also been identified, a true major target of neurodegeneration may be the intracellular signaling machinery provided by the kinome [5]. Kinases are known to play critical roles in various cell signaling pathways [6] and have been confirmed to be dysregulated in a number of diseases, including neurodegeneration [7]. They provide a link between cell surface recognition events triggered by the binding of cell adhesion molecules, extracellular matrix, or other soluble factors (e.g., growth factors) and intracellular signaling pathways in neuronal cells [8]. Based on the observations of an inverse relationship between cancer and ND, a focus has been placed in the past on some of the cancer kinases to target intracellular signaling pathways at the intersection between the control of cellular metabolism and proliferation, inhibition of which was thought to halt neurodegeneration [5], but so far this inhibition has not shown an efficient therapeutic effect [9,10]. Nonetheless, therapeutics targeting kinases currently still account for approximately 50% of anticancer drug discovery efforts [7].

Non-receptor protein tyrosine kinases of the Src family (c-Src, c-Fyn, c-Yes, and c-Abl) are associated with ND as they are involved in axonal and dendritic outgrowth during central and peripheral nervous system development and regeneration [8]. In this context, aberrant c-Abl activation was shown to cause early neuroinflammation and loss of neurons in the forebrain of Niemann–Pick type C (NPC) transgenic mice [11], and increased c-Abl activation has been reported in neurodegenerative pathologies of PD, AD, ALS and FTD by us and others [11,12,13]. In the brains of patients with AD, c-Abl activity is associated with the formation of neuritic plaques and insoluble neurofibrillary tangles [14], whereas in FTD-FUS cases, increased c-Abl activity was associated with C-terminal Tyr phosphorylation of FUS protein and its aggregation in cortical neurons [12]. This suggests that abnormal activation of c-Abl may contribute to nonspecific posttranslational modifications of ND-related proteins, which may then promote the occurrence of features associated with ND, such as the accumulation of insoluble protein aggregates and impaired mitochondrial function, both of which are accompanied by synaptic damage.

**Figure 1:**
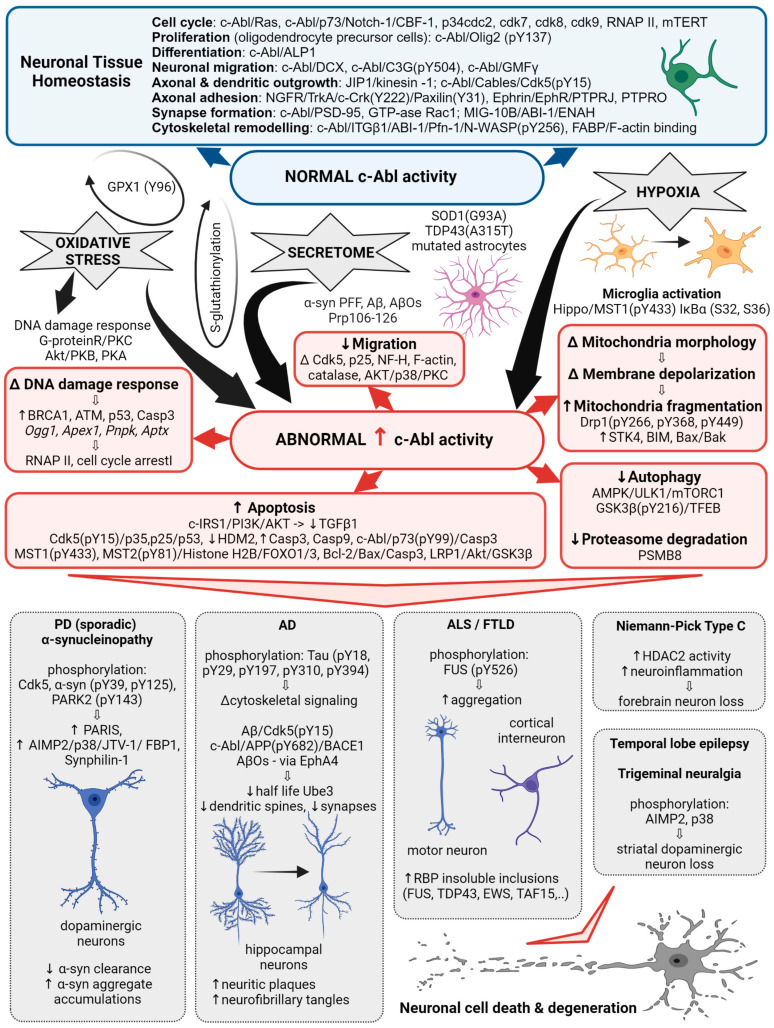
c-Abl signaling involved in multiple cellular processes. Schematic illustrating the different Abl signaling pathways discussed in the following text and highlighting the correlation with the abnormal processes associated with neurodegenerative diseases.

Oxidative stress, the most likely trigger of abnormal kinome activation, has long been implicated in the pathogenesis of ND [15] and has been reported as a major cause of sporadic PD [16], where it is responsible for much of the dopaminergic neuronal damage [17]. The ubiquitously expressed non-receptor tyrosine kinase c-Abl is activated by oxidative stress and plays a role in oxidative stress-induced neuronal cell death [18,19]. There it is even considered an indicator of oxidative stress [16,17]. Selenocysteine insertion sequence-associating factors, adenosine, Arg kinase and c-Abl kinase are all potent Se-independent regulators of expression and activity of glutathione peroxidase-1 (GPX1) gene/protein, which plays a protective role in neuronal cells in coping with oxidative damage [20]. Activation of c-Abl, with few exceptions, mostly negatively affects enzymes involved in antioxidant defense. Still, c-Abl can be modified by S-glutathionylation, and this reversible modification leads to the down-regulation of its kinase activity [11,21]. Inversely, depending on the oxidation level in the cell, glutathione peroxidase can also be activated via phosphorylation at Tyr96 by c-Abl [22]. Although the constitutively active form of c-Abl, Bcr-Abl, has a long history in myeloid and lymphoblastic leukemia, aberrant activation of c-Abl has emerged as a link between various triggers of oxidative stress relevant to PD, AD, FTLD and α-synucleinopathies [12,15,23]. Inhibition of c-Abl kinase activity by small molecule compounds used in the clinic to treat human leukemia showed neuroprotective effects in cell and animal models of PD [24]. Unfortunately, to date, several c-Abl kinase inhibitors have shown only sub-threshold efficacy in clinical trials [9,10], most likely due to limited knowledge of c-Abl signaling. Therefore, here we review the functions and effects of c-Abl in neuronal cells discovered to date to demonstrate how different aspects of c-Abl signaling contribute to the progression of neurodegenerative diseases. See Figure 1 and the following sections for an explanation.

## 2. The Structure of c-Abl and Its Role in Neurodegenerative Diseases

Initially, the non-receptor tyrosine kinase c-Abl was identified as a protooncogene activated in a subset of human leukemias [25], yet quite soon it was associated with neurodegeneration. The c-Abl kinase has a complex structure consisting of multiple domains and motifs that are also found in other signal-transducing proteins [25] and have been reviewed in detail elsewhere [26]. Protein-protein interaction screens of a phage expression library have identified proteins that interact with specific domains of c-Abl and can be termed regulators or effectors of c-Abl activity. In this way, SH3-domain-containing proteins, amphiphysin-like protein 1 (ALP1) and amphiphysin are proposed to interact with the c-Abl carboxyl terminus to regulate its role in cell differentiation in vitro and in vivo [25]. Expression of ALP1 leads to the morphological transformation of NIH 3T3 fibroblasts in a c-Abl-dependent manner that involves remodeling of the cytoskeleton [25]. Moreover, increased c-Abl activity was detected in oligodendrocyte progenitor cells, which are essential for myelination during central nervous system development. In these, c-Abl-mediated phosphorylation of the transcription factor Olig2 was confirmed indispensable for the proliferation of oligodendrocyte progenitor cells [27]. Yet the homology of the yeast proteins Rvs167 and Rvs161 with the amino terminus of c-Abl and abnormal activation of c-Abl also suggest that it is involved in cell cycle arrest [25], neuroinflammation [28], and may cause neuronal death via activation of apoptotic signaling pathways [29]. Table 1 summarizes the status of c-Abl expression and activity detected in ND.

In PD, abnormally increased c-Abl activity is associated with the accumulation of pathogenic α-synuclein (α-syn) [30]. Increased expression and activation of c-Abl has been found in mouse models of PD and AD and in neuronal cultures in response to inclusions formation and oxidative stress. Overexpression of active c-Abl in mouse neurons leads to neurodegeneration and neuroinflammation [31]. Levels and activity of c-Abl are greatly increased in the brain tissue of patients with PD [32,33,34]. In dopaminergic neurons, this is accompanied by increased phosphorylation of c-Abl protein substrates, such as α-syn and the E3 ubiquitin ligase, parkin [24,35]. In animal models, the use of different c-Abl inhibitors has been shown to improve motor behavior in animals and prevent loss of dopaminergic neurons [1,35], while the inhibitors Nilotinib and Radontinib even showed improvement in motor and cognitive symptoms in PD patients [35]. The expression of c-Abl is increased in trigeminal neuralgia, where it is accompanied by the loss of dopamine neurons in the striatum via aminoacyl-tRNA synthetase-interacting multifunctional protein type2 (AIMP2, p38) activation [28]. In PD models, c-Abl inhibitors reduce phosphorylation of Cdk5, decrease phosphorylation and clearance of α-syn and parkin, and decrease levels of several parkin substrates such as zinc finger protein 746 (PARIS), AIMP2, fuse-binding protein 1 (FBP1), and synphilin-1 [17,32,36]. Radotinib has even been demonstrated to protect primary cortical neurons from toxic cell death induced by c-Abl activation with preformed α-syn fibrils and to reduce Lewy bodies/Lewy neurites-like pathology [35]. Overall, increased activation of c-Abl through parkin inactivation, accumulation of its toxic substrate AIMP2, α-syn aggregation, and impaired autophagy are shown to be associated with neurodegenerative processes of PD.

In AD, c-Abl plays a role in the development of Tau pathology by regulating cytoskeletal signaling cascades. Immunocytochemical studies show that c-Abl is associated with both neuritic plaques and neurofibrillary tangles in the brains of patients with AD. c-Abl interacts directly with Tau and phosphorylates it at tyrosine 394 [23,36], which has a regulatory effect on normal Tau-related processes, including microtubule assembly and axonal transport, and to trigger aggregation of Tau into paired helical filaments [26]. Neuronal spine pathology is associated with the early onset of AD. Amyloid beta oligomers (AβOs) are known to induce synaptotoxicity, leading to synaptic dysfunction/loss and the reduction in dendritic spine density that underlies cognitive defects [4]. c-Abl was activated in neurons exposed to AβOs and in the brains of patients with AD. Inhibition of active c-Abl ameliorated all AβOs-induced synaptic changes [37] and cognitive deficits in the AD mouse model [4]. AβOs induction of c-Abl signaling appears to involve the tyrosine kinase ephrin receptor A4 (EphA4) [4] and decreases the number of mushroom spines in c-Abl knockout neurons, while preserving the populations of immature stubby, filopodia spines, suggesting that c-Abl deficiency increases the population of immature spines and decreases AβOs-induced synapse elimination [37].

**Table 1:** c-Abl expression and activity in disease.

Status	Detected in Patients and Cell or Animal Models	Reference
Increased c-Abl expression	PD and AD patients’ brains (neuritic plaques, neurofibrillary tangles) dying substantia nigra dopaminergic neurons accumulating a-Syn neurons, oligodendrocytes with α-Syn aggregates in transgenic mice glial cytoplasmic inclusions in MSA patients temporal neocortex of patients with temporal lobe epilepsy degenerating forebrain neurons in the CA1 region of the hippocampus, neuronal loss preceded by substantial microgliosis and astrocytosis in AblPP/tTA mice double cortex, lissencephally where disrupted neuron migration Niemann–Pick type C patients’ brains differentiating myoblasts (myogenesis) developing rat hippocampus neurons (postsynaptic compartment)	[11,15,23,24] [38,39] [9,40] [9] [41] [23,31] [42] [43] [44] [45]
Increased c-Abl activity	PD patients’ brains, neurons of substantia nigra and striatum AD patients’ brain neurons FUS-FTLD patients’ cortical brain neurons striatum in C57bl/6J mice, primary neuron culture several Gaucher mouse disease models stress induced primary cortical neurons dopaminergic neurons of striatum (trigeminal neuralgia model) stressed dopaminergic neurons of α-Syn A53T Tg mice forebrain neurons in mouse neuroinflammation model temporal neocortex of patients with temporal lobe epilepsy brain neurons of subarachnoid hemorrhage rat model ABO treated HT22 hippocampal neuron cells 1-Methyl-4-phenylpyridinium iodide induced rat midbrain neuron	[32,34,46,47] [48] [12] [47] [49] [50] [28] [30] [11] [41] [51] [52] [33]
c-Abl deficiency	c-Abl KO neurons increase dendritic spine density c-Abl KO mice exhibit reduced α-syn aggregation, neuropathology, neurobehavioral deficit conditional c-Abl KO mice exhibit decreased parkin phosphorylation	[37] [40] [46]

In ALS, increased c-Abl expression was found in motoneurons [13]. A phenotypic screen of motor neurons derived from induced pluripotent stem cells (iPSCs) from ALS patients with SOD1 mutation revealed that more than half of the tested drugs that stop neuronal cell death target the Src/c-Abl pathway. The Src/c-Abl inhibitors increased the survival of iPSC-derived motor neurons from ALS patients in vitro, and siRNA knockdowns of *c-Src* or *c-Abl* prevented their degeneration. Likewise, Bosutinib inhibitor increased in vitro survival of iPSC-derived motor neurons from patients with sporadic or familial forms of ALS, caused by mutations in the TAR DNA binding protein (TDP-43) or repeat expansions in the *C9orf72* gene [53]. Moreover, conditioned media from primary mouse astrocytes expressing either mutant human SOD1(G93A) or TDP43(A315T), but not from SOD1(WT) astrocytes, increased c-Abl activity in rat motoneurons, interneurons and glial cells in vitro, which was detected 60 min after exposure, and resulted in neuron death within days. This effect of the conditioned media was prevented by the use of the c-Abl inhibitor Imatinib, blockers of Na channels (spermidine, mexiletine, or riluzole), and antioxidants (Trolox, esculetin, or tiron) [3].

Finally, increased c-Abl activity has been found in other ND as well. This way increased c-Abl activity was noted in cortical neurons of FTLD-FUS patients [12], whereas both total and phosphorylated c-Abl were found upregulated in the temporal neocortex of patients with temporal lobe epilepsy compared to nonepileptic controls [41]. In the temporal neocortex of model rats treated with pilocarpine, upregulation of total and phosphorylated c-Abl begins 6 h after seizures, with relatively high levels persisting for 60 days, whereas in the hippocampus, elevated c-Abl levels persist for 30 days after seizures and then return to normal [41]. Given the evidence of c-Abl activation in the brain of patients with various ND, improved targeting of its signaling may indeed prove advantageous for novel treatment designs.

## 3. The Role of c-Abl in Brain Injuries

In addition to ND, c-Abl also appears to promote neuronal cell death in brain injury caused by hypoxia and cerebral ischemia [51]. In particular, cerebral ischemia-reperfusion injury represents a major public health problem that causes high rates of disability and death in adults [54]. Under hypoxic conditions, the normal development and migration of brain cells can be severely impaired, and c-Abl appears to be involved in processes leading to cell death. For example, increased c-Abl protein levels were observed in rat pups exposed to intermittent hypoxia during embryonic development, resulting in a delay in neuronal migration early in the postpartum period. The downstream targets of c-Abl: Cdk5, p25, and the cytoskeletal elements neurofilament H, F-actin, and catalase were all found altered [55]. Likewise, computational analyses of phosphoprotein datasets covering the response of sensory neurons to axonal injury identified 400 redundant axonal signaling networks, among which the signaling hub proteins c-Abl, AKT, p38, and protein kinase C, were overrepresented [56]. Moreover, endogenous c-Abl protein levels and neuronal apoptosis also increase 24 h after subarachnoid hemorrhage. This could be inhibited with c-Abl inhibitors decreasing cleavage of caspase-3 and enhancing the phosphorylation of Akt and glycogen synthase kinase (GSK)3β [51].

Activation of microglia also plays a role in the alteration of the neuronal microenvironment caused by ischemic stroke. c-Abl has been found to be involved in the mechanism underlying microglial activation and subsequent oxidative stress-induced cell death of primary neurons. c-Abl was found to phosphorylate Hippo/MST1 protein kinase at Y433, which increases its activity for phosphorylation of IκBα at residues S32 and S36, and lead to microglial activation [54]. Oxidative stress in brain cells can also be unknowingly induced by the use of iron-oxide nanoparticles as therapeutics or for supplemental intake in iron deficiency. Apparently, 24-h exposure of human SH-SY5Y neuroblastoma cells to 10 μg/mL of 10- and 30-nm iron oxide nanoparticles not only decreases cellular dopamine content by 50% but also increases and activates c-Abl and neuronal α-Syn expression. In mice exposed to these nanoparticles, the number of active mitochondria in neuronal cells and striatal dopamine and its metabolites decrease, and neuropathological damage to neuronal cell bodies, dopaminergic terminal, and neuronal vasculature occurs [57].

## 4. The Effects of c-Abl Activity on Neuronal Cells

### 4.1. Synaptic Plasticity

In addition to its role in cell cycle regulation and apoptosis, c-Abl is also involved in cytoskeletal remodeling, a process that is critical for normal central nervous system development [31] including neuronal migration, neurite outgrowth, and synaptic plasticity [41]. It is the impairment of synaptic plasticity accompanied by synapse loss, that characterizes the early stages of ND [58,59]. Active c-Abl is present in brain synapses, although its precise synaptic function is still unknown. In rats, the c-Abl protein levels increase postnatally in the hippocampus, with c-Abl expression peaking in the first postnatal week and in 14-day cultured hippocampal neurons. There, c-Abl is found primarily in the postsynaptic compartment, where it colocalizes with the postsynaptic scaffold protein, the so-called postsynaptic density protein-95 (PSD-95), which is critical for synapse formation [45]. Conversely, a breakpoint cluster region (BCR)—a Rac GTPase-activating protein known to form a fusion protein with c-Abl in Philadelphia chromosome-positive myeloid leukemia—is found abundantly expressed in the brain and localizes to excitatory synapses, where it interacts with PSD-95. Since chemical and genetic inhibition of c-Abl activity showed a reduction in PSD-95 tyrosine 533 phosphorylation and an increase in PSD-95 clustering and synapse formation [45], it has been suggested that excessive activity of Rho family small GTPase Rac1 negatively affects synaptic and cognitive function and leads to mental retardation in AD [60]. There is one study though claiming that c-Abl deficiency in cAbl knock-out neurons increases dendritic spine density and synapse formation, yet these results remain isolated [37]. In the Tg2576 mouse model of AD, increased Aβ oligomers levels were shown to activate c-Abl and decrease the half-life of the neuron-specific Ube3A protein, the degradation of which precedes the age-dependent behavioral deficits and loss of dendritic spines in these mice [59]. AβOs can also activate c-Abl in dendritic spines of cultured hippocampal neurons via EphA4 receptor tyrosine kinase, leading to synaptic loss, dendritic spine degradation and neuronal cell death that could be prevented by the antagonistic EphA4 peptide KYL and the c-Abl inhibitor Imatinib [58]. The effects of numerous c-Abl inhibitors are summarized in Table 2.

Finally, some other chemotrophic factors also seem to impair the c-Abl-mediated synaptic function. One of them, netrin, can initiate different neurodevelopmental programs in individual neurons in vivo, via MIG-10 isoforms localizing to specific subcellular domains. In this way, the MIG-10B isoform localizes uniquely to presynaptic regions and induces synaptic vesicle clustering in response to netrin. There, it interacts with Abl-interacting protein-1 (ABI-1), a component of the WAVE complex that regulates ABI-1/c-Abl-mediated phosphorylation of ENAH, which is critical for organizing the actin cytoskeleton at presynaptic sites and directing vesicle clustering through SNN-1/synapsin at the membrane [79]. This docking of synaptic vesicles at the plasma membrane may be disrupted by extensive α-synuclein phosphorylation in PD. There, excessive phosphorylation of α−syn at Tyr39 by c-Abl facilitates the conversion of synuclein from the vesicle-bound extended-helix state to the broken-helix state, presumably disrupting the fusion of synaptic vesicles with the plasma membrane [80]. Therefore, in the developing embryonic brain, the activation of c-Abl appears tightly regulated, so as to not intervene negatively with dendrite growth, branching, and the number of synapses formed.

### 4.2. Cytoskeletal Dynamics and Cell Migration

Cytoskeletal dynamics and cell migration are the next processes associated with c-Abl activity that are involved in the development of neuronal polarity. Before primary cortical neurons become polarized, c-Jun N-terminal kinase (JNK)-interacting protein-1 (JIP1) localizes specifically to a single neurite and accumulates in the emerging axon. There, phosphorylated c-Abl kinase interacts with JIP1 and promotes axonal growth through its binding to kinesin-1 [81]. In brains, c-Abl also phosphorylates cyclin-dependent kinase 5 (Cdk5), which interacts with Cables [82]. Cables also interact with c-Abl and enhance c-Abl-mediated Cdk3 and Cdk5 Tyr15 phosphorylation [83]. Silencing of Cables inhibits neurite growth in primary cortical neurons, whereas increased expression of active c-Abl results in neurite elongation [82], and inhibition of c-Abl/CABLES/p-CDK5 signaling by Dexibuprofen prevents impairment of spatial learning and memory loss in a transgenic AD mouse model [75].

Next, c-Abl is involved in cytoskeletal dynamics, because the expression of amphiphysin-like protein 1 (ALP1) led to the morphological transformation of NIH 3T3 fibroblasts in a c-Abl-dependent manner [25]. Human c-Abl has an F-actin binding domain (FABD) and its activity is inhibited by direct F-actin binding, although various physiological signals that regulate the actin cytoskeleton can activate it. The c-Abl induction of actin microspike formation in fibronectin-spreading fibroblasts depends on its kinase activity and is not shared by c-Src kinase activity. The c-Abl-dependent F-actin microspikes develop only under conditions where Rho-family GTPases are inhibited. Similarly, FABD-mutated c-Abl, which is active in detached fibroblasts, stimulates F-actin microspikes independently of cell attachment and stimulates also the formation of F-actin branches in neurites of rat cortical neurons. This reciprocal F-actin/c-Abl regulation is thought to provide a self-limiting mechanism for controlling actin cytoskeleton dynamics [84]. At the molecular level, c-Abl has been shown in flies to negatively regulate the actin cytoskeleton effector protein Ena during neuronal development. There, c-Abl binds via an SH2 domain to Lamellipodin (Lpd), which regulates cell motility by recruiting Ena/VASP proteins (Ena, Mena, VASP, EVL) to the leading edge of cells. Phosphorylation of Lpd by c-Abl appears to be required for the interaction between Lpd and Ena/VASP proteins. Both netrin-1 and platelet-derived growth factor (PDGF) stimulate phosphorylation of Lpd by c-Abl in primary cortical neurons and promote axonal morphogenesis and PDGF-induced dorsal ruffling [85].

In addition, c-Abl also appears to control the axonal motility, and adhesion via the Crk family of adapter proteins, which are known to regulate anchorage-dependent DNA synthesis and cytoskeletal reorganization [86]. Nerve growth factor (NGF) is known to promote dimerization of the tyrosine kinase receptor TrkA and phosphorylation of c-Crk and paxillin proteins. c-Abl is recruited to the NGF receptor complex, where it interacts with TrkA and c-Crk proteins [87]. In this signaling cascade, c-Abl appears as a regulator of NGF-induced c-Crk phosphorylation at Tyr(222), which is followed by Tyr(31) phosphorylation of paxillin and dissociation of the SH2 domain of Crk from paxillin and the SH3 domain of Crk from c-Abl. This phosphorylation cycle seems crucial for this multiprotein complex turnover and cytoskeletal dynamics [86]. Remodeling of the microtubules is together with actin dynamics required for axon guidance [88]. Eph receptors have been shown to play a role in axon guidance of retinal ganglion cells at the optic chiasm. There c-Abl acts downstream of ephrin-Eph signaling for the repulsion of retinal axons at the optic chiasm, by being a substrate for protein tyrosine phosphatase receptor type J and O (PTPRJ and PTPRO). PTPRJ regulates the guidance of retinal axonal projections by controlling the ephrin-Eph-c-Abl axis [89]. Similar studies in Drosophyla and Xenopus models suggest that c-Abl acts as a central signaling node to coordinate actin and microtubule dynamics downstream of the guidance receptors. There, c-Abl has been shown to interact with a microtubule-associated protein Orbit/MAST that mediates the action of Slit and its receptors [88].

Tight regulation of cell motility is essential for normal neural development [85], with many malformations recognized as a cause of mental retardation and epilepsy. In the pathological condition known as double cortex (DC), an abnormal band of neurons is found in the white matter underlying a normal cortex. A doublecortin (DCX) gene has been found to encode a cytoplasmic protein with c-Abl and MAP-kinase phosphorylation sites, suggesting that DCX and c-Abl are crucial for the migration of developing neurons [42]. A Rap1 guanine nucleotide exchange factor (C3G) has been shown to be involved in cell adhesion and migration, and with active c-Abl required for C3G (Tyr504 phosphorylation)-induced reorganization of actin cytoskeleton and filopodia formation in migrating cells. However, although C3G interacts with c-Abl and its overexpression leads to enhanced localization of c-Abl in the cytoplasm, they both seem to function in an interdependent manner when linking external signals to cytoskeleton remodeling and filopodia formation [90,91]. Conversely, TGFβ response in proliferating neurofibromas that involves hyperactivation of a Ras/c-Abl pathway, increases collagen synthesis and inhibits cell motility [71]. The aforementioned Abl-interacting protein 1 (Abi1), also plays a role in cell migration, where it localizes to the tip of lamellipodia and coordinates with F-actin at the leading cell edge of migrating cells. Integrin β1 and c-Abl regulate the recruitment and positioning of Abi1 at the leading edge, where Abi1 regulates motility by affecting Pfn-1 and N-WASP [92,93]. c-Abl also phosphorylates glia maturation factor-γ (GMFγ) to increase focal adhesion dynamics and migration. A phosphomimetic mutant, Y104D-GMFγ, was found enriched along the leading edge, where it recruits activated N-WASP (pY256) to promote the actin-branch formation, enhancing lamellipodial dynamics and limiting focal adhesions [94].

## 5. Direct Association of c-Abl with Amyloid Proteins

Whether c-Abl activity is directly involved in the formation of toxic intracellular protein inclusions remains to be confirmed, but its activation is almost always detected in degenerating neurons. Oxidative stress is a major cause of sporadic Parkinson’s disease (PD), and either external or internal triggers of oxidative stress can both activate c-Abl and lead to neuronal death [16,74]. An increased activation of c-Abl along with increased α-syn phosphorylation has been detected in α-syn expressing mice, in glial cells (oligodendrocytes, astrocytes) and neurons with cytoplasmic inclusions [9]; as well as in substantia nigra neurons and in cerebrospinal fluid from PD patients [33,34,38,95]. Overexpression of α-syn in substantia nigra neurons activates c-Abl through a redox stress mechanism [38]. Also, exposure to preformed α-syn fibrils (PFFs) induces oxidative stress and c-Abl activation in wild-type mouse neurons, whereas α-syn- deficient neurons, which cannot form α-synuclein aggregates, do not exhibit c-Abl activation. α-Syn aggregates thus induce c-Abl activation, with activated c-Abl then, in turn, promoting α-syn phosphorylation and aggregation, in a feed-forward interaction [36,74]. Once active, c-Abl kinase interacts directly with α-syn and catalyzes its phosphorylation at Tyr39 and to a lesser extent at Tyr125 [39,95], which downregulates α-syn clearance and promotes its aggregation leading to cell death [9,39,40]. Age-dependent increase of phospho-Tyr39 α-syn has also been detected in the brains of healthy individuals, whereas c-Abl has been found upregulated only in the brains of PD patients. Moreover, mice expressing a human α-syn mutation (hA53Tα-syn mice), or in which c-Abl has been knockdown, show reduced α-syn aggregation, neuropathology, and neurobehavioral deficit [40]. The complete reduction of α-syn aggregate burden in the striatum cortex and in the substantia nigra was thus not observed in mice treated with c-Abl inhibitor Nilotinib, which reduced α-syn phosphorylation only by 40% [9]. But Nilotinib has been shown to enhance α-syn autophagic clearance via enhanced deposition of α-syn into the lysosomes and to partially improve motor performance [38]. Nonetheless, c-Abl plays a role in α-syn-induced neurodegeneration and joint inhibition of c-Abl and some downstream effectors may prove neuroprotective.

Autosomal recessive PD is also caused by mutations in PARK2/parkin, which encodes a ubiquitin E3 ligase responsible for the ubiquitin tagging of proteins for degradation [46]. Oxidative and dopaminergic stress activates c-Abl in cultured neuronal cells and in the striatum of C57BL/6 mice, and activated c-Abl was found in the striatum of PD patients [47]. C-Abl regulates the neuroprotective functions of parkin by phosphorylating parkin at Tyr143, which inhibits its ubiquitin E3 ligase activity [34,39,46]. Loss of ubiquitin ligase activity of parkin leads to accumulation of the parkin substrates AIMP2 (p38/JTV-1) and FBP1 and cell death in the substantia nigra and striatum of PD patients [46,47]. In contrast to Nilotinib, the c-Abl inhibitor Imatinib, completely prevents phosphorylation of parkin and maintains the cytoprotective function of parkin [34,46]. Conditional knockout of c-Abl in mouse neurons also completely prevents phosphorylation of parkin, accumulation of its substrates, and drug-induced neurotoxicity [46]. Likewise, a second-generation irreversible c-Abl kinase inhibitor, INNO-406, is capable of preventing dopaminergic neuronal death in a toxin-induced mouse model of PD, due to its more efficient blood-brain barrier transfer [17].

The c-Abl kinase also phosphorylates tau, which is an important microtubule-associated protein of axons and forms paired helical filaments (PHFs) that make up the neurofibrillary tangles found in AD [36,96,97]. Human tau has five tyrosines numbered 18, 29, 197, 310, and 394, corresponding to the sequence of the longest CNS isoform. Tau with phosphorylated tyrosines 18, 197, and 394 was found in the brains of patients with AD, whereas only tau with phosphorylated Tyr394 was found in healthy humans [48]. C-Abl kinase directly phosphorylates tau at Tyr394, which was found in PHF [48,96,98]. Arg, the other member of the Abl family of tyrosine kinases, can phosphorylate tau at Tyr394 independently of Abl kinase activity [98], and Tyr18 of tau appears to be phosphorylated by Fyn kinase as well [96]. Recently, some controversial evidence was presented. Phosphorylation of either all five tyrosine residues, several N-terminal tyrosine residues (Tyr18, 29, and 197), or specific phosphorylation of only residue Tyr310 has been shown to abolish tau aggregation and inhibit its microtubule- and lipid-binding properties. NMR analyses suggest that these effects are mediated by a local decrease in the β-sheet propensity of paired helical filament PHF6 domain of tau [97]. It is hypothesized that hyperphosphorylation of tau is more likely to contribute to neurodegeneration of AD through microtubule destabilization [48], with c-Abl activity still shown to be critical for neurodegeneration in tauopathies.

In AD, amyloid-β-peptide (Aβ) activates c-Abl [63,64], which phosphorylates tau and Cdk5 at Tyr15 [62]. Levels of active c-Abl and tau phosphorylation are therefore increased in Aβ-treated mouse neurons and could be normalized by treating APPswe/PSEN1ΔE9 transgenic mice with Imatinib inhibitor [62]. Brains from patients with Niemann–Pick type C disease (NPC) also exhibit elevated Aβ-peptide levels and increased c-Abl activity. In AD animal models, c-Abl interacts directly with the cytosolic domain of Alzheimer’s amyloid precursor protein (APP), and Tyr682 phosphorylation in the cytoplasmic tail of APP is essential for APP-BACE1 interaction and promotion of amyloidogenic processing of APP to Aβ-oligomers and the carboxy-terminal fragment βCTF [43,73]. Imatinib reduces APP-BACE1 interactions and APP amyloidogenic cleavage in cells and brains of AD and NPC mouse models and also reduces cognitive deficits [43,61]. Aβ and AβOs activate c-Abl in neurons [4,43,61,63,64,99] and blocking c-Abl activity rescues neurons from cell death in mammals and Drosophyla. In Drosophyla neuronal cells, active c-Abl appears to be required for Cdk5 binding, activation, and translocation, but unlike in humans, is not required for the conversion of p35 to p25, suggesting that Cdk5 activation and translocation are independent of p25 in the Drosophila model of AD [99].

## 6. Gene Expression and DNA Damage Response

Kinases are also associated with the regulation of gene expression and response to DNA damage, which appear as a major cause of many ND. The integrated cytoplasmic and nuclear functions of c-Abl suggest a mechanism through which signaling initiated at the plasma membrane may determine the response to corticosterone in neurons via the transcriptional machinery regulated by classical nuclear mineralocorticoid, and glucocorticoid receptors. Signal transduction from a membrane G protein-coupled receptor can activate PKC, Akt/PKB, and PKA and subsequently trigger phosphorylation of the tyrosine kinases Pyk2, c-Src, and c-Abl [100], which can trigger transcriptional repression of synaptic genes via downstream mechanisms [52]. This way in NPC neurons, increased c-Abl kinase activity in turn increases HDAC2 phosphorylation and activity and suppresses the expression of key synaptic genes. Inhibition of c-Abl by methyl-β-cyclodextrin and vitamin E conversely prevents HDAC2 activity and recruitment to the synaptic gene promoters, allowing synaptic gene expression [77]. Similarly, in AD, repression of neuronal gene expression is induced by enhanced c-Abl activity, through increased HDAC2 repression activity, whereas this is decreased in c-Abl knockout cells [52]. c-Abl also mediates neuronal cell death via the interaction of HDAC3 and c-Fos in mouse models of Huntington’s disease, where *c-Fos* expression is selectively reduced in the striatum and its forced expression has been shown to protect against the neurotoxic effects of activated c-Abl and HDAC3 [101].

c-ABl also appears to be involved in cell cycle regulation, with transcription factor p73 implicated in neurodevelopment and maintenance of the mature central nervous system. Endogenous p73 has been shown to be stabilized by c-Abl and to antagonize Notch-1 intracellular domain/CBF-1-dependent gene transcription and differentiation of SH-SY5Y neuroblastoma cells [102]. Haploinsufficiency of p73 causes neuronal death, possibly through activation of the tau-related kinases c-Abl, GSK3β, and Cdk5, in the brains of aged p73^+/−^ mice, in which aberrant phospho-tau-positive aggregates form, suggesting that p73 is required to protect mouse brains from hyperphosphorylation of tau during aging [103]. In addition to affecting transcription factor binding, c-Abl also functions with cyclin-dependent kinases, including p34cdc2, cdk7, cdk8, and cdk9, in cell cycle-dependent modification (phosphorylation) of the carboxyl-terminal domain of RNA polymerase II (RNAP II) that affects RNAP II transcriptional activity [104]. c-Abl also interacts directly with mTERT and downregulation of c-Abl decreases mTERT expression, which is associated with decreased proliferating cell nuclear antigen (PCNA) expression [105].

DNA damage has been linked to the pathogenesis of neurodegenerative diseases such as ALS and AD [104,106]. However, the exact relationship between DNA damage accumulation, DNA damage response (DDR), and neuron vulnerability in ALS and AD remains unclear. DNA damage (single-stranded DNA gaps and 8-hydroxy-deoxyguanosine incorporation) probably accumulates in ALS motor neurons along with decreased DDR, as DNA repair genes undergo hypermethylation [106]. Different types of DNA repair counteract highly toxic DNA double-strand breaks (DSBs) to maintain genome stability. DDR in ALS motor neurons results in the accumulation of activated c-Abl, nuclear BRCA1, ATM, p53, and cleaved caspase-3, as well as hypomethylation of the DNA repair genes *Ogg1*, *Apex1*, *Pnkp*, and *Aptx* [106]. In DDR, c-Abl and BRCA1 are recruited to the nucleus, initiating the repair of DNA DSB in ALS motoneurons [106]. At DNA lesions, c-Abl phosphorylates RNAP II and causes stalling of RNAP II, which stimulates p53 accumulation and leads to cell cycle arrest. Similarly, AD pathology is also thought to be due to activation of mitotic Cdks and c-Abl, and phosphorylation of RNAP II in postmitotic neurons [104]. Recent evidence suggests that DDR utilizes small RNA species produced as long non-coding (nc)RNA precursors that recognize DSBs. At the DSB, c-Abl kinase causes the formation of RNAPII foci, which trigger the synthesis of strand-specific damage-responsive transcripts (*DART*s) there. This then triggers the formation of double-stranded (ds)RNA intermediates via DNA-RNA hybrid intermediates to promote the recruitment of p53-binding protein 1 (53BP1) and Mediator of DNA damage checkpoint 1 (MDC1) to DSBs [107]. The c-Abl phosphorylation of DDR proteins and impairment of their expression can potentially attenuate DART synthesis and cause a delay in DNA damage repair in neurodegeneration.

Activation of c-Abl kinase by DNA damage may also trigger oligomerization of the unfolded protein response transducer IRE1α, which controls the stability of mRNAs involved in DDR through endoribonuclease activity and catalyzes their IRE1α-dependent decay (RIDD) [108]. c-Abl also regulates the activity of the transcription factor MyoD and Pax7 by direct phosphorylation during the DDR and pauses cell differentiation [44]. In irradiated cells, the intermediate filament synemin appears to be an upstream positive regulator of c-Abl activity in DSB repair, as silencing of *Synm* causes the deactivation of several tyrosine kinases, including c-Abl. Synemin binds to the SH2 domain of c-Abl and allows hyperphosphorylation of c-Abl at Tyr412 and Thr735 in an ATM kinase-dependent manner [109]. ATM kinase also activates c-Abl by phosphorylating it at Ser 465 in response to ionizing radiation [110]. Moreover, inactivation of ATM proves to act antiapoptotic in differentiated neurons, whereas inactivation of c-Abl proves to act antiapoptotic only in immature neurons, in cell culture, and in the cerebral cortex in vivo [111]. DSB in cortical neurons therefore induce rapid p53-mediated apoptosis through actions of upstream ATM/c-Abl kinases and downstream mitochondrial death proteins, but these depend on neuronal maturity [111]. Finally, phosphorylation of Mdm2 by c-Abl and ATM regulates Mdm2-p53 signaling and targeting of p53 to proteasomal degradation [112]. The DDR, in which c-Abl plays a role [104,106,107,108], might normally act neuroprotectively to block S-phase-dependent apoptosis induction [113]. This may allow cell cycle events to be sustained in vivo in affected neurons for weeks to years before apoptosis is observed.

## 7. c-Abl and Autophagy

Impairment of the autophagy-lysosomal pathway leads to the accumulation of misfolded proteins and dysfunctional organelles, observed in neurodegenerative diseases such as PD and AD [2,30,33]. In AD model Tg2576 mice, dopaminergic neurons of the ventral tegmental area undergo functionally relevant changes at the onset of degeneration associated with autophagosome accumulation, suggesting dysfunctional autophagy accompanied by increased activation of c-Abl kinase [2]. Treatment of AD model Tg2576 mice with nilotinib reduces c-Abl phosphorylation/activation and enhances autophagy, leading to a reduction in Aβ levels and preventing degeneration of dopaminergic neurons [2]. With regard to the ubiquitin-proteasome pathway in proliferating cells, silencing of the Abl gene and activation/inhibition of c-Abl kinase activity have been shown to affect expression levels of the proteasome subunit beta-type 8 precursor (PSMB8) and its alternatively spliced isoforms [114].

In PD, the c-Abl kinase is activated by cellular stress and promotes α-syn pathology either by direct phosphorylation of α-syn, as previously described, or by inhibition of autophagy [30]. Overexpression of α-syn in mouse and rat brains enhances c-Abl expression, and interestingly, the Nilotinib inhibitor has been shown to induce α-syn protein degradation via autophagy and proteasome pathways [39,40]. In the α-syn mouse model (TgA53T) and in human PD cases, c-Abl activation is accompanied by increased p53 activation. Active p53 in TgA53T neurons accumulates in the cytosol and inhibits autophagy, leading to α-synucleinopathy. Treatment of these neurons with Nilotinib and PD180970 inhibits c-Abl, increases autophagic flux and α-syn clearance by inducing phosphorylation of AMP-activated kinase (AMPK), ULK1 activation, and downregulation of mammalian target of rapamycin complex 1 (mTORC1) signaling [30,78]. Active c-Abl also directly interacts with GSK3β and catalyzes its phosphorylation at Tyr216, which then negatively regulates transcription factor EB (TFEB) and decreases its nuclear translocation, leading also to impaired autophagy [33]. TFEB—a master regulator of lysosomal biogenesis, exocytosis, and autophagy is therefore negatively regulated by c-Abl mediated phosphorylation [72]. Inhibition of c-Abl with Imatinib promotes lysosomal biogenesis, autophagy, and exocytosis by facilitating nuclear translocation of TFEB and promoting expression of its target genes independent of mTORC1 signaling in cells and animal models of PD [33,72]. In NPC disease models characterized by the accumulation of free cholesterol in lysosomes [115], inhibition of c-Abl promotes cholesterol clearance in a TFEB-dependent manner [72]. Another c-Abl inhibitor, Bosutinib, has been shown to boost autophagy in iPSC-derived motor neurons, reduce the amount of misfolded mutant SOD1 protein, and attenuate mitochondrial gene expression [53], altogether directly pointing to the exact role of c-Abl not only in intracellular aggregates formation but also in their clearance.

## 8. c-Abl and Mitochondria

Oxidative stress has been identified as one of the causes of ND. Mitochondria have been implicated in the oxidative stress response, as stress-induced mitochondrial dysfunction leads to neuronal death in neurodegenerative diseases [116]. They play a crucial role in reactive oxygen species (ROS)-mediated pathways, and several gene products related to mitochondrial function are the subject of neurodegeneration research [116]. Toxic factor(s) released from ALS-mutant genes expressing astrocytes can trigger hyperexcitability and increased calcium influx, affecting mitochondrial structure and physiology in neuronal cells [3]. ROS production, mediated in part by changed mitochondrial metabolism, is known to trigger c-Abl signaling. When primary rat spinal cord cultures are exposed to conditioned media derived from primary mouse astrocytes expressing human SOD1(G93A), Nav channel-mediated excitability and calcium influx increase, intracellular ROS are generated, and motoneurons death occurs within days. Only the conditioned medium from hSOD1(G93A) but not SOD1(WT) astrocytes increases c-Abl activity and death in motoneurons, interneurons, and glial cells, which could be inhibited by the c-Abl inhibitor Imatinib. SOD1(G93A) conditioned medium induces changes in the morphology of neuronal mitochondria related to their membrane depolarization. Blocking the opening of the mitochondrial permeability transition pore with cyclosporine A or inhibiting mitochondrial calcium uptake with Ru360 reduces ROS production and c-Abl activation [3]. Among other proteins, c-Abl mediates phosphorylation of Dynamin-related protein 1 (Drp1), a known regulator of mitochondrial dynamics, at Tyr266, Tyr368, and Tyr449, which increases its GTPase activity in vitro and in vivo and promotes Drp1-mediated mitochondrial fragmentation and death of primary cortical neurons [50].

However, oxidative stress and mitochondrial changes may not only be the triggers of the ND but may also act as accelerators of ND, as the appearance of toxic protein aggregates and insoluble inclusions may further promote changes in mitochondrial morphology and its fragmentation. In prion diseases, pathogenic accumulation of misfolded prion proteins (e.g., the scrapie form of PrP9) leads to activation of c-Abl and mitochondrial apoptotic signaling, resulting in neuronal death, prevented by Imatinib. A synthetic neurotoxic prion fragment (PrP106-126) has been shown to activate c-Abl, which in turn triggers upregulation of STK4 and BIM activation of Bax/Bak lipid pore permeability. Further changes in mitochondrial membrane potential lead to complete mitochondrial dysfunction with abrupt Bax translocation to mitochondria and release of cytochrome c into the cytosol. This together with the activation of caspase-9 and caspase-3 leads to neuronal death [117]. There are other protective proteins in mitochondria whose function may also be altered in ND. PTEN-induced kinase 1 (PINK1) protects cells from mitochondrial dysfunction and its mutations are associated with the autosomal recessive familial form of PD. In addition to c-Abl in neurons, PINK1 is also induced by oxidative stress, and its mitochondrial network of genes includes the E3 ubiquitin ligase Parkin, the protease presenilin-associated rhomboid-like serine protease, the protein kinase MARK2, the protease HtrA2, and tumor necrosis factor receptor-associated protein 1 (TRAP1) [116]. Most often though, the impairment of mitochondrial function leads to programmed cell death or apoptosis of neuronal cells.

## 9. c-Abl and Apoptosis

In causing neuronal cell death, c-Abl has been associated with cell cycle control [23,41,62] and cell division processes involving high TERT telomerase activity [118]. Both c-Abl and mTERT were found expressed during the prenatal and postnatal development of mouse reproductive organs [118]. Consistently, c-Abl was found to be highly expressed in cancer tissues and cells, exhibiting aggressive growth and proliferation [119]. Depletion of c-Abl results in the inhibition of proliferation and an increase of apoptosis in several cell lines (SW480, HCT-116, etc.) through inhibition of TGF-β1 signaling via the IRS1/PI3K/AKT pathway [119]. However, in terminally differentiated brain neurons, increased expression of active c-Abl leads to severe neurodegeneration. Neuronal loss is preceded and accompanied by substantial microgliosis and astrocytosis. Since c-Abl expression has never been found in glial cells, this suggests that preferably neuronal c-Abl expression is responsible for the observed gliosis [23]. Namely, c-Abl expression is upregulated threefold in sporadic ALS spinal motor neurons that undergo apoptosis, compared to non-ALS spinal motor neurons [13]. Also, many viruses, such as enterovirus EV71 (causing neurological deficits in children), induce neuronal apoptosis via activation of c-Abl and Cdk5 kinase, whereas it does not affect c-Abl and Cdk5 activities in non-neuronal cells [69]. A mutation of Cu/Zn superoxide dismutase-1 (SOD1) present in familial ALS has been shown to upregulate c-Abl, activate caspase-3, and promote apoptosis in motor neurons from G93A transgenic mice [13].

In oxidative stress, the tumor suppressor p53 stimulates growth arrest and apoptosis in response to DNA damage [120]. The activity of p53 is controlled at transcriptional, translational, and post-translational levels. Under oxidative stress, c-Abl modulates the activity of Cdk5 via phosphorylation of Tyr15 and in cooperation with cleavage of p35 to p25. Cdk5 increases the stability of p53 through its post-translational modification. Together, c-Abl and Cdk5 regulate maximal activation of p53, leading to neuronal apoptosis [30,121]. p53 interacts with HDM2, an E3 ubiquitin ligase, leading to nuclear export and degradation of p53. Stress-induced c-Abl down-regulates HDM2, leading to an increase in p53 levels [121]. p53 drives apoptosis of immature and differentiated mouse embryonic cortical neurons post-DSB induction by upregulating mitochondrial death proteins [111]. Hyperglycemia also increases the expression of c-Abl and formation of p53-cAbl complex in neural progenitor cells and drives them to apoptosis in vitro, whereas causing defects in neural-tube formation in vivo [122].

Retinoblastoma tumor suppressor protein (RB) is a downstream effector in p53-mediated cell cycle arrest that inhibits E2F and nuclear c-Abl. Namely, E2F activates transcription of *p73* mRNA and c-Abl stabilizes p73 protein, a p53 homolog and activates its pro-apoptotic function [120]. The accumulated Aβ fibrils can activate c-Abl kinase in mice and rats, which then by phosphorylating p73 (nuclear c-Abl/p73 complex) and increasing expression of apoptotic genes leads to neuronal apoptosis [63,64]. In APPsw/PSEN1DeltaE9 AD mice, increased c-Abl activation, p73 phosphorylation, tau phosphorylation, and caspase-3 activation in neurons surrounding Aβ deposits are associated with cognitive decline [63]. The human immunodeficiency virus type 1 (HIV-1) trans-activator of transcription (Tat) protein is known to similarly deregulate neuronal functions via the p73 and p53 pathways. Tat uses microRNA-196a to promote p73phosphorylation at Tyr99 by c-Abl, but this does not lead to neuronal cell death due to parallel hyperphosphorylation of the RB protein [123]. Patients with Niemann–Pick type C disease, characterized by accumulation of free cholesterol and glycosphingolipids in the endosomal-lysosomal system, exhibit progressive loss of Purkinje neurons in the cerebellum. Cholesterol accumulation and oxidative stress activate apoptosis in Purkinje neurons in NPC model mice via the proapoptotic c-Abl/p73 signaling [67,115]. Surprisingly, infusion of Angiotensin II (Ang II), a vasoactive substance of the renin-angiotensin system, known to function in vasoconstriction, aldosterone release, and cell growth was shown to induce a substantial incidence of AD symptoms in vivo via c-Abl activation. Overexpression of c-Abl enhances Ang II-induced apoptosis, whereas inhibition of c-Abl attenuates Ang II-induced apoptosis in mice [70]. Ang-II-mediated apoptosis requires the AT2 receptor and causes the release of cytochrome *c* from mitochondria with concomitant activation of caspase-3 and DNA fragmentation, suggesting mitochondrial-mediated apoptosis, without the involvement of the extrinsic apoptotic pathway, and only caspase-9, but not caspase-8, activation [124].

Mammalian Ste20-like kinases (MSTs) are the homolog of Drosophila hippo and play a critical role in regulating stress-induced cell death of mammalian primary neurons and astrocytes [65,125]. c-Abl phosphorylates MST1 at Tyr433 and MST2 at Tyr81 within the kinase domain [66,125]. Phosphorylation of MST2 disrupts the interaction with Raf-1 proteins and leads to homodimerization of MST2, which thereby enhances MST2 activation and triggers neuronal cell death [125], whereas c-Abl phosphorylation of MST1 at Tyr433 directly stabilizes and activates MST1 [66]. Activated MSTs exert a pro-apoptotic function through cleavage, autophosphorylation, and in turn phosphorylation of downstream targets such as MST1/Histone H2B and Forkhead box O1/3 (FOXO1/3) [66,125], leading to neuron death in primary culture and in the rat hippocampus [65]. Inhibition of c-Abl promotes degradation of MST1 through the C-terminus of Hsc70-interacting protein (CHIP)-mediated ubiquitination and attenuates cell death [65]. Silencing of *Mst1* also inhibits the induction/activation of c-Abl [66], suggesting that the two kinases are regulated by a reciprocal activation mechanism.

Finally, c-Abl also plays a role in injury-induced neuronal apoptosis via Bcl-2/Bax/Casp3 [76], and by regulating the LRP-1-dependent Akt/GSK3β survival pathway [51]. The active, phosphorylated form of c-Abl is increased in models of Gaucher disease, where it interacts with RIPK3, phosphorylating it and leading to cell death [49].

## 10. Inflammatory Processes Regulation in Neurodegeneration

Inflammatory processes may act as triggers or accelerators of neuronal degeneration. Based on studies in microglia and neuronal cultures, as well as experiments in animal models and the clinical findings, a number of endogenous and external signals could activate microglial cells [54,126] and induce NF-kappa-beta with the consequent release of inflammatory cytokine mediators such as TNFα, IL-6 and IL-1β. Overexpression of these mediators triggers signaling cascades in neurons leading to activation of protein kinases GSK3β, Cdk5, and c-Abl, and inactivation of phosphatases such as PP1, resulting in hyperphosphorylation and self-aggregation of proteins in ND [126]. The loss of neurons in ND is preceded and accompanied by significant microgliosis and astrocytosis [127]. When c-Abl is activated by oxidative stress in an acute PD model, p38α was identified as the main substrate of c-Abl, and c-Abl-mediated phosphorylation was found to be crucial for the dimerization of p38 (AIMP2) [16], which in turn initiates the expression of IL-1, IL-6, TNFα. Neuroinflammation is common to α-synucleinopathies and tauopathies; where it is associated with c-Abl and neurodegeneration. An aged mouse model of α-synucleinopathy harboring human mutant A53T α-Syn shows increased c-Abl activation and tau phosphorylation along with age-related changes in immunity, associated with loss of IL-10, decreased levels of CCL2, CCL5, IL-2, and IL-3, when compared to young A53T mice. Nilotinib and Bosutinib c-Abl inhibitors, can both reduce α-Syn and p-Tau in the brain and periphery and affect neuroinflammation by altering several immune blood markers except IL-10 and CX3CL1 [36]. Systemic sclerosis is an idiopathic autoimmune disease that besides other non-receptor tyrosine kinases also involves c-Abl [128]. Phosphorylation of mitochondrial antiviral signaling protein (MAVS) by c-Abl appears to be required for the death of dopaminergic neurons during brain inflammation [129].

Similarly, interferon (IFN)-induced activation of the signal transducer and activator of the transcription (STAT) family appears to be mediated by c-Abl. The non-receptor kinases c-Abl and Arg interact directly with STAT1 and mediate phosphorylation of STAT1 at Tyr701 independently of Janus kinases and in the absence of IFNγ. The presence of IFNγ potentiates c-Abl-mediated STAT1 phosphorylation, STAT1 dimerization, nuclear translocation, and downstream inflammatory gene transcription [130]. Consistently, two of the signaling pathways upregulated in Abl-PP/tTA mice with increased c-Abl expression were cell cycle and interferon signaling. Increased expression and activation of STAT1 is an early consequence of c-Abl activation in Abl-PP/tTA mice and occurs in the hippocampus prior to neurodegenerative pathologies and neuroinflammation development [127]. In addition to the IL/TNFα signaling pathway, c-Abl /STATS mediated transcription of pro-inflammatory genes can also be activated by other receptors during neurodegeneration. In the dog model of human autoimmune demyelinating disease alterations were thus noted besides c-Abl also in PDGFR-α, PDGFR-ß, and c-Kit signaling [131]. At last, c-Abl has been shown to regulate vascular barrier integrity [132]. The c-Abl (*Abl1*) and the Abl-related gene (*Arg*, *Abl2*), phosphorylate cytoskeletal effectors that mediate vascular permeability, including non-muscle myosin light chain kinase, cortactin, vinculin, and β-catenin, and in this way regulate the dynamics of cell-cell and cell-matrix junctions [132]. Overall, though it appears that the inflammatory processes is triggered via different pathways, all pathways are associate with c-Abl signaling at some point; therefore, even from this perspective, a more thorough search for improved c-Abl kinase inhibitors could prove beneficial

## 11. Summary

Nonreceptor tyrosine kinases, particularly c-Abl, indeed appear to be involved in a variety of processes that when out of balance can lead to neuronal death. Nevertheless, several c-Abl inhibitors developed in clinical trials to date have shown disappointing efficacy. The answer probably lies in the fact that there are many more signaling networks present in vivo than under in vitro conditions or in animal models, and we all keep forgetting that mice are not yet humans. It is also important to keep in mind that c-Abl is only one of the many kinases involved in normal brain function as well as in ND. For example, post-mortem AD and PD brains show that the levels of several other tyrosine kinases are elevated there and also the knockdown of these tyrosine kinases was shown to reduce aggregation of neurotoxic proteins, including α-synuclein, β-amyloid and tau [133]. Thus, we can conclude that a multi-kinase target that includes c-Abl and other tyrosine kinases could offer more benefits in alleviating neurodegenerative pathologies than a target that is selective for c-Abl only. There is an urgent need to integrate knowledge at the level of different cellular processes as exemplified by this review. Only the performance of additional studies that elucidate the role of the kinome and possible synergistic and antagonistic actions of multiple kinome members in early brain pathological changes in neurodegenerative diseases can provide a rational basis for new therapeutic interventions.

## Figures and Tables

**Table 2:** The effects of c-Abl inhibitors in cell and animal models.

Inhibitor Disease	Model	Outcome of Inhibitor Use	Refs
**Nilotinib**			
PD	Transgenic α-Syn mice	40% ↓ α-syn phosphorylation in the striatum and cortex	[9]
	PD patients	↑ motor and cognitive functions	[35]
	Mouse expressing Abl, α-Syn in substantia nigra	↓ Abl activity, ↑ clearance of α-Syn by autophagic degradation ↑ lysosomal deposition, ↑ neurons survival, ↑ motor performance	[38]
	A53T a-syn mouse	↑ autophagic flux, ↓ accumulation of a-Syn, delayed disease onset	[30]
	Mouse exposed to MPTP	↓ Abl activation, ↓ levels of PARIS ↓ DA neuron loss and behavioral deficit	[34]
AD	DA neurons (VTA) in Tg2576 mouse overexpressing human APP695	↑ autophagy, ↓ autophagosome accumulation ↓ Aβ levels ↑ DA outflow to hippocampus ↓ hippocampal associated cognitive deficit	[2]
	α-Syn expressing mice, striatum, cortex neurons	↓ Abl activity 30–40% ↓ α-Syn phosphorylation, no change of α−Syn aggregate burden	[9]
AD, NPC	NPC neurons AD transgenic mice	↓ APP amyloidogenic cleavage in neurons overexpressing Aβ and APP ↓ Aβ burden in brains of AD mouse model	[43]
**Nilotinib Bosutinib**			
α-Syn Tauopathy	Old A53T mice (human A53T α-Syn)	↓ brain and peripheral α-Syn and p-Tau, modulated blood immunological response, altered microglia morphology, ↓ astrocytes and dendritic cells	[36]
**Bosutinib**			
ALS	SOD1 iPCS MN SOD1 mice	↑ survival of motor neurons in vitro, ↑ autophagy, ↓ misfolded SOD1 ↓ expression of mitochondrial genes, ↑ survival of SOD1 mice	[53]
**Imatininb (Imatinib mesylate, Gleevec, STI-571)**			
PD	SN4741 cells, primary midbrain neurons	↑ autophagy-lysosomal pathway, ↑ nuclear translocation of TFEB	[33]
	Neuronal cells (striatum) of C57BL/6 mice	↓ parkin phosphorylation ↓ accumulation of substrates AIMP2, FBP1	[47]
	Neuronal cells, parkin (+), cAbl cond. KO mouse	↓ parkin phosphorylation ↑ catalytically active and protective parkin	[46]
	Mice exposed to short term MPTP treatment	↓ Abl activity ↓ loss of DA neurons, ↓ locomotive defects	[16]
AD	Transgenic AD mouse overexpressing Aβ, cAbl KO mice, ABO expressing cells,	↓ AβO in plasma, ↓ AD brain plaques and AβO accumulation ↓ neuroinflammation and cognitive deficits ↓ levels of beta-CTF fragments	[61]
	Neurons Transgenic AD mouse expressing AβO	↓ HDAC2 levels ↓ repression activity and HDAC2 recruitment to the promoter of synaptic genes	[52]
	Neurons in culture AD mouse (AβO)l	↓ dendritic spine reduction ↓ neuronal apoptosis by AβO	[58]
	APPswe/PSEN1ΔE9 mouse, primary neurons	↓ Tau phosphorylation that is induced by Aβ activation of c-Abl/cdk5	[62]
	APPsw/PSEN1DeltaE9 transgenic rat model	↓ cABl/p73 signaling, ↓ rat behavioral deficit induced by Aβ, tau phosphorylation and apoptosis	[63]
	Rat hippocampal neurons expressing Aβ	↓ neuronal cell death	[64]
Oxidative stress induced ND	P6 rat cerebellar granule neurons, E18 rat embryo hippocampus neurons exposed to H_2_O_2_, rotenone treated rats	↓ oxidative stress induced c-Abl autophosphorylation ↓ downstream MST1 phosphorylation ↓ cell death	[65]
	Primary cortical astrocytes treated H_2_O_2_	↓level of FoxO1/3 and Mst1 ↓ cell death	[66]
NPC	NPC1 (Niemann–Pick type C) mouse	↑ survival of Purkinje neurons, ↓ apoptosis in cerebellum, due ↓ c-Abl/p73 signaling	[67]
Prp(Sc)	Neuro2A cells, rat hippocampal neurons of E18 embryos, both tretaed with Prp106-126 peptide	↓ c-Abl kinase activity ↓ MMP change, ↓ Bax translocation to mitochondria ↓ cytochrome c release ↓ activation of BIM expression	[68]
enterovirus EV71 infect	Non neuronal cells	↓ c-Abl and Cdk65 activation ↓ neuronal apoptosis in cells infected with EV71	[69]
Trigeminal neuralgia	Infraorbital nerve ligation TN rat model	↓ P38 expression ↓ loss of DA neurons	[28]
VSMC degeneration	AngII infused mouse, Vasc. smooth muscle cell	↓ AngII-induced apoptosis ↓ phenotypic transformation of VSCM in vivo and in vitro	[70]
Neurofibromas	NF1+/− fibroblasts Fibroblasts from neurofibromas	↓ excessive collagen synthesis ↓ proliferation ↓ Ras-cAbl signaling and TGF-β mediated fibroblast recruitment	[71]
**Imatinib with Spermidine, Mexiletine, Riluzol**			
ALS (SOD1)	Rat spinal cord cells exposed to SOD1(G93A), SOD1 (wt) Ms astrocytes	↓ c-Abl activity ↓ motoneuron death mediated through mitochondrial alterations	[3]
**Imatinib with antioxidants (troloc, esculetin tiron)**			
ALS	Rat spinal cord cells exposed to SOD1(G86R), TDP43(A315T) astrocytes	↓ motor neuron death	[3]
**Imatinib, nilotinib, dasatinib, GNF-2, analog GNF-5**			
NPC (Niemann–Pick type C)	HeLa TFEB-GFP cells HT22 TFEB-GFP cells HEK293 TFEB-GFP cells	↑ TFEB nuclear translocation, ↑ TFEB activity ↓ c-Abl activity ↑ lysosomal exocytosis and autophagic flux, ↑ cholesterol clearance	[72]
**Dasatinib**			
ALS (SOD1)	Mouse SOD1 motor neurons Transg. G93A-SOD1 mice	↓ c-Abl phosphorylation, ↓ neuronal cytotoxicity ↓c-Abl phosphorylation, ↓ caspase-3 ↑ innervation status of neuromuscular junctions	[13]
**Radotinib**			
PD	Primary cortical neurons, α-Syn pre-formed fibrils (PFF) inj. C57BL/6 mouse	↓ neuronal toxicity, ↓ α-Syn PFF induced c-Abl activation ↓ dopaminergic neuron loss neuroinflammation and behavioral deficits ↓ β-Syn PFF induced toxicity in mice	[35]
**EGCG—epigallacatechin gallate**			
Amyloidosis	MC65 neuronal cells expressing Aβ fragment APP-C99	↓ levels of AB, ↑ APP nonamyloidogenic proteolytic processing ↓ nuclear translocation of c-Abl ↓ c-Abl/Fe65 interaction	[73]
**N-acetyl cysteine**			
PD	PFF exposed Wt neurons, AAV-mediated α-Syn overexpressing mouse	↓ PFF-induced c-Abl activation in wt neurons, ↓ α-Syn aggregation ↓ dopaminergic neuronal loss ↓ microglia activation and motor impairment	[74]
**Dexibrufen (DXB)**			
AD	APPswe/PS1dE3 mice	↓ activation of glial cells, ↓ cytokine release (TNFα), ↓ soluble Aβ plaque deposition, ↑ Aβ degradation ↓ Tau hyperphosphorylation, ↓ c-Abl/CABLES/Cdk5 signaling ↓ memory impairment, ↑spatial learning	[75]
**INNO-406, second generation Abl inhibitor**			
PD	C57bl/6 mouse exposed to toxic MPTP	↓ c-Abl phosphorylation of parkin, ↓ AIMP2 accumulation, ↓progression of DA neuronal damage	[17]
**LY294002**			
SAH	Subarachnoid hemorrhage mice model	↓ neuronal apoptosis, mortality and neurological deficits ↓ expression of cleaved caspase 3	[51]
**Propofol**			
animal model	Propofol treat. rat brains	↓ c-Abl expression and ROS, ↑ neuronal survival	[14]
**Levo-tetrahydropalmitine (L-THP)**			
Cer. I/R injury	Cerebral ischemic rats	↓ c-Abl expression, ↓ neuronal apoptosis in injured rats	[76]
**Methyl-beta-cyclodextrin, vitamin E, + Two inhibitors**			
NPC	NPC neuronal models, Npc1−/− mice	↓ c-Abl/HDAC activation in NPC neurons, Npc1−/− mice, ↓ HDAC2 recruitment to promoter, ↓ neuronal genes’ expression	[77]
**Small molecule inhibitor PD180970**			
PD	HeLa cells, dopaminergic N27 cells, microglial BV2 cells, MPTP exposed mice	↑ autophagy in an mTOR-independent manner ↓ α-Syn toxicity in cells and mice ↓ microglial activation	[78]

Table symbols: ↑—increased, ↓—decreased.

## Data Availability

No new data were created in this study. All the data used are contained within the article.
